# A Robot-Based Tool for Physical and Cognitive Rehabilitation of Elderly People Using Biofeedback

**DOI:** 10.3390/ijerph13121176

**Published:** 2016-11-24

**Authors:** Leire Lopez-Samaniego, Begonya Garcia-Zapirain

**Affiliations:** eVida/DeustoTech-LIFE Lab., University of Deusto, Bilbao 48007, Spain; leire.lopez@opendeusto.es

**Keywords:** sensors, biofeedback, serious games, Bluetooth LE

## Abstract

This publication presents a complete description of a technological solution system for the physical and cognitive rehabilitation of elderly people through a biofeedback system, which is combined with a Lego robot. The technology used was the iOS’s (iPhone Operating System) Objective-C programming language and its XCode programming environment; and SQLite in order to create the database. The biofeedback system is implemented by the use of two biosensors which are, in fact, a Microsoft band 2 in order to register the user’s heart rate and a MYO sensor to detect the user’s arm movement. Finally, the system was tested with seven elderly people from La Santa y Real Casa de la Misericordia nursing home in Bilbao. The statistical assessment has shown that the users are satisfied with the usability of the system, with a mean score of 79.29 on the System Usability Scale (SUS) questionnaire.

## 1. Introduction and Background

During recent years, life expectancy in developed societies has been increasing at an unprecedented rate [1] and this tendency is likely to continue. The ageing population is both one of man’s greatest achievements and challenges in the 21st century. Furthermore, due to higher birth rates in developed societies in the last decades, a great percentage of the population will belong to the third age by the middle of this century. These predictions have boosted research in the field of healthy ageing with the aim of making the future elderly more independent.

In order to prevent early ageing and delay the natural ageing of elderly people, “active ageing” and “healthy ageing” are being tested. These concepts refer to staying active during one’s lifetime, performing activities that boost different skills such as physical [2], psychic and social well-being in order to extend productivity, quality of life and life expectancy to advanced ages. These activities include daily actions such as walking, jogging, writing, solving “Sudokus” or mathematical problems etc.

Such activities can and are being adapted to technological solutions and can now be performed through the use of devices such as tablets and smartphones. It must be noted that these devices give access to many different activities, wherever the patient is, and even make them easier than they were originally.

Furthermore, there are other technologies that are, in fact, promising and useful for cognitive and motor rehabilitation. These include technologies such as wheelchairs to detect and correct problems with the objective of improving people’s quality of life. One of these technological solutions is an “intelligent carpet”, which is able to detect the footsteps and provide information about them to help caregivers to create routings to strengthen leg muscles, to improve balance etc. [3], and a patch which, when combined with a sensor system, detects the user’s movements in order to know if the user has fallen and whether to alert the family. 

Another promising technology is Assistive Robotics (AR), which has led to Socially Assistive Robotics (SAR) and merges the previously mentioned traditional activities and social interaction with AR [4,5,6,7,8]. Indeed, these technologies are of great interest as different studies have stated that they may increase user motivation by providing visual feedback. The objective of SARs is to provide some form of close and effective interaction with the user. Many projects can be found in this area, such as the Maggie robot [9], which is able to perform multiple activities like reading a book to blind people, helping elderly people move or even acting as a playmate. There is another project being carried out by the University of Southern California, whose purpose is to provide instructions, evaluation and to encourage users to perform arm exercises [10,11,12].

Serious games and Information and Communication Technology (ICT)-based solutions are also a research area for cognitive and physical rehabilitation. One such example is a home-based physical rehabilitation game for tablets, developed by researchers from Hasselt University in Belgium [13] or a pilot study carried out by Eindhoven University in the area of both physical and cognitive rehabilitation designed for stroke patients [14]. Furthermore, there are other ICT-based applications, whose potential users are not elderly people, but which also try to boost both physical and cognitive rehabilitation of different groups, such as children with cerebral palsy (CP), people with Down syndrome, etc. [15,16,17,18].

Lastly, biological sensors and biofeedback systems are being exponentially merged with these types of systems both for cognitive and physical telerehabilitation programs in order to make it possible to adapt the rehabilitation and maintenance program to each patient’s needs [19,20,21]. It is noteworthy that there are not many existing systems in this field, as it is a quite young area in which research is now beginning. However, some systems can be found, such as the UniTherapy project [22], which via interaction between the therapy provider, the user and the intervention of a rehabilitation system, can change the performance level of the activity, depending on the user’s expertise, their disability level, task execution level and the performance of previous activities. Hence, this is the field of study discussed in this article. This study presents a system that is capable of measuring the heart rate with a Microsoft band in order to reduce or increase the level of difficulty of the game depending on the user’s biological state. Furthermore, the use of an electromyography (EMG) sensor will allow the user to train both cognitively and physically with the same game due to the need to control the game with arm movements.

Bearing this in mind, the main objective and the specific objectives of this project are described as follow:

- General objective: Design and implementation of a Lego-based robot for both cognitive and physical maintenance and rehabilitation of elderly people, taking into account biological parameters regarding arm movements and heart rate values.
- Specific technological objectives:
  (1) Design and implementation of the communication between the Lego-based robot EV3 and an iPad mini.
  (2) Design and implementation of the serious game.
  (3) Data acquisition from biological sensors.
  (4) Validation of the system with real patients.

## 2. Methods

This section provides an in-depth description of the materials and the methodology used in the development of the system in addition to the description of the pilot testing sample.

### 2.1. Design of the Platform

**Figure 1:**
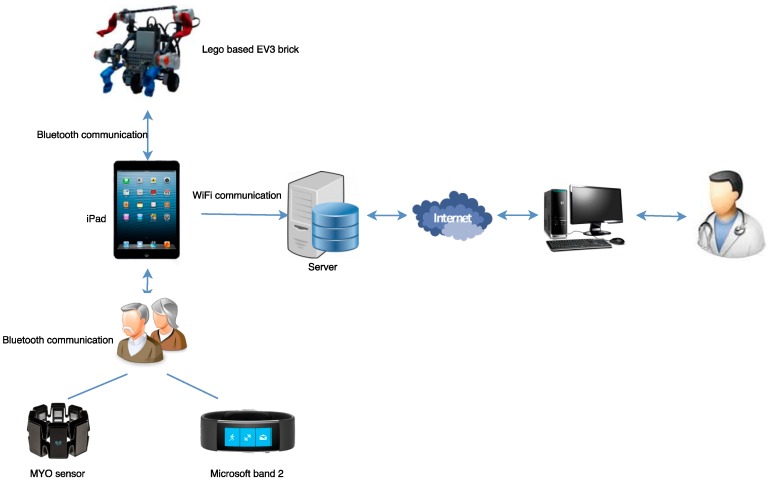
Architecture of the system.

Section 2.1 provides a complete description of the system design, whose general architecture is summarized in Figure 1. As shown, the iPad features an iOS (iPhone Operating System) application containing the serious games which the user will interact with. Furthermore, performance of the serious game will be conditioned by the data received from two sensors: the MYO (Thalmic Labs, Canada) and the Microsoft band (Microsoft Corp., Redmon, WA, USA). It must be noted that both sensors are connected to the iPad through a Bluetooth Low Energy (Bluetooth LE) connection. These serious games contain both different cognitive and physical activities, taking into account that the physical activities are accompanied with the use of a Lego-based robot, by the integration of the Lego EV3 brick (The Lego group, Denmark). Finally, all the data gathered by the iPad, both the scores obtained in the games and biological data, are locally stored and sent to a server, which will be accessible to the nursing home staff through a website.

**Figure 2:**
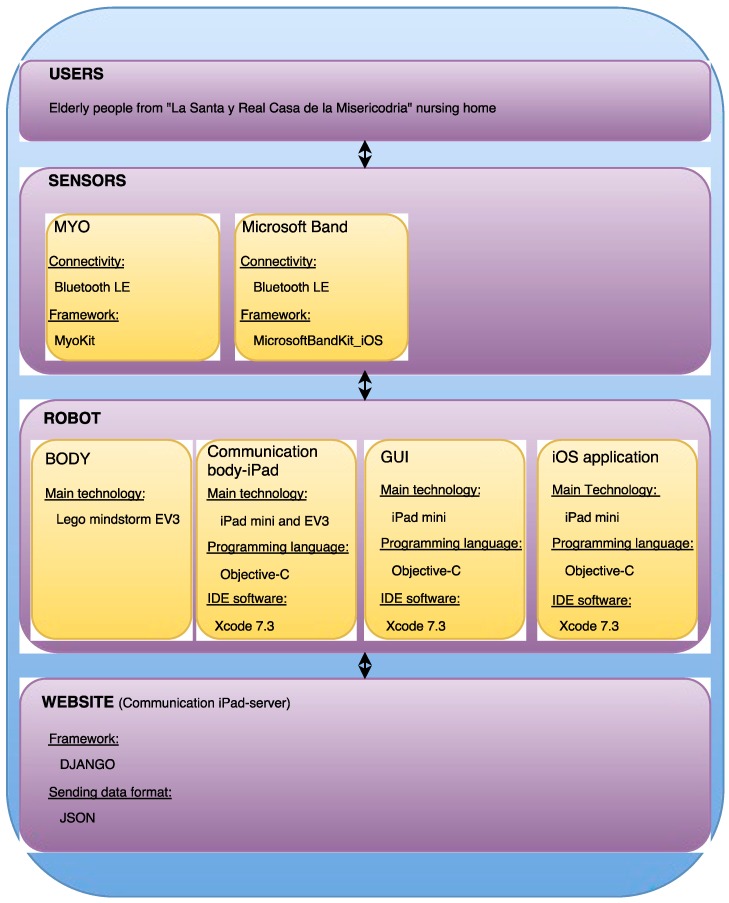
High-level design of the system.

Figure 2 shows the high-level design of the whole system, presenting in detail the information about the connectivity protocol the devices used, the frameworks, the main technologies, programming languages and IDE (Integrated Development Environment) software that have been used throughout development of the system.

As shown in Figure 1, the design of the entire system consisted of four main stages:

(1) The design of the Graphical User Interface (GUI) of the iOS application, as well as the serious game that the users from La Santa y Real Casa de la Misericordia have to play.
(2) The design of the biofeedback, consisting in the following two sub-stages:
  (2.1) The design of the wireless communication between the iPad mini and the biological sensors.
  (2.2) The design of the biofeedback system itself to track how activities change depending on the values received.
(3) Communication between the Lego EV3 brick and the program which will be run on the iPad.
(4) Communication between the iPad and the server and the website accessible to the nursing home staff.

#### 2.1.1. Stage 1: Graphical User Interface and Game Run on the iOS Application

The main objective of the first stage is to design the graphical interface of the iOS application and the serious games that the application will host. Given the fact that the potential users of this system are elderly people and the high likelihood of visual dysfunction, this stage is extremely important. In fact, the system should ensure large legible labels, buttons and text fields. Furthermore, there should be a marked contrast between the different colors so that they can be easily distinguished by the vast majority of the users.

In addition to the graphical interface, this stage covers the serious game design. This game covers a brain reflexes activity for the cognitive rehabilitation, combined with the MYO sensor. This combination allows the user to control the game through arm movements and, hence, including motor rehabilitation. As mentioned before, the iOS application hosts the graphical user interface which shows the cognitive and physical game being run. Furthermore, if the exercise is properly performed, the Lego robot makes movements to motivate the elderly. Communication between the iOS device and the EV3 brick will take place in stage number 4. 

Finally, this stage also covers the design of the SQLite database. In fact, this is necessary in order to provide personalized biofeedback and to perform the log-in processes.

#### 2.1.2. Stage 2: Biofeedback

Stage number 2 will fully describe both the design of the communication protocol between the iPad and the biological sensors as well as the design of the biofeedback system.

#### 2.1.3. Stage 3: Data Acquisition of the Biological Sensors

The main objective of this substage is to design the communication protocol between the MYO EMG, the Microsoft band and the iOS device for the acquisition of the biological data. In fact, the design of the biofeedback response is covered in stage number 2.2.

It has to be taken into account that the communication protocol will vary depending on the sensor. In fact, data acquisition from the Microsoft band will be carried out by the usage of the “MicrosoftBandKit_iOS” framework (Microsoft Corp., Redmon, WA, USA) whereas data acquisition from the MYO sensor will be done though the “MyoKit” framework provided by Thalmic Labs (Canada).

**Figure 3:**
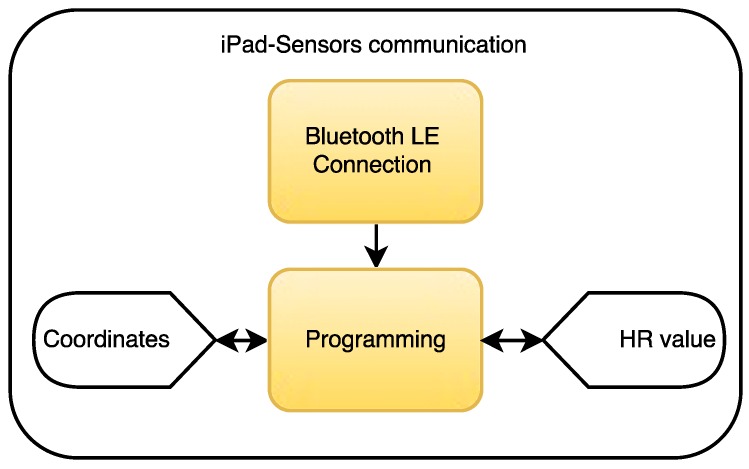
Data acquisition protocol from the MYO sensor and Microsoft band. (HR = Heart Rate).

As shown in Figure 3, the iPad will receive position coordinates from the MYO sensor and the heart rate values from the Microsoft band. It has to be taken into account that the MYO will detect change in movement at any time, while the Microsoft band will detect the heart rate once every second.

#### 2.1.4. Stage 2.2: Design of the Biofeedback System

As stated before, stage 2.2 describes the design of the biofeedback system, which is the way in which the system will react, based on the biological data that have been received. 

On one hand, the serious game designed has to be introduced. It consists of a brain reflexes game. According to the level, the player will have to play against one or two enemy balls. The user will control the football either with his or her finger or with the use of the MYO sensor. If the user has a MYO sensor, it will detect each movement and will have to avoid colliding with the basketballs. In fact, by using this sensor, the user performs not only cognitive exercises but also physical movements.

**Figure 4:**
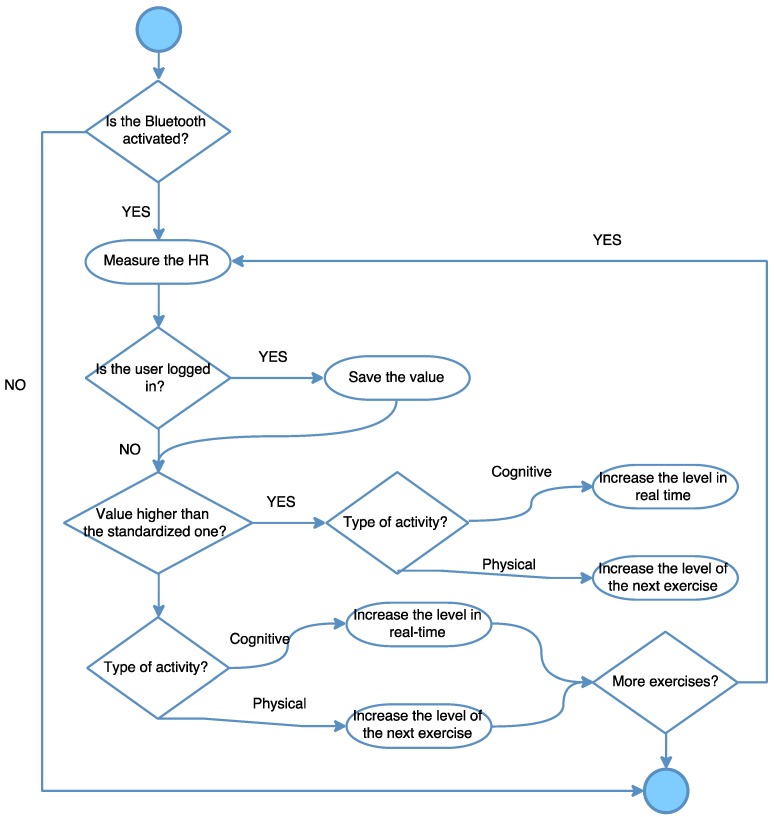
Flowchart diagram of the heart rate (HR).

On the other hand, as shown in Figure 4, if the Bluetooth LE is activated, the system will begin to measure the data every second. In the case that the user is logged in, the system will save the results to the local database. If the value is higher than the standardized ones, the activity will immediately decrease its difficulty or, in contrast, if the value is lower than expected, the system will increase the difficulty of the activity.

#### 2.1.5. Stage 3: Communication with the EV3 and Programming of the Activities

**Figure 5:**
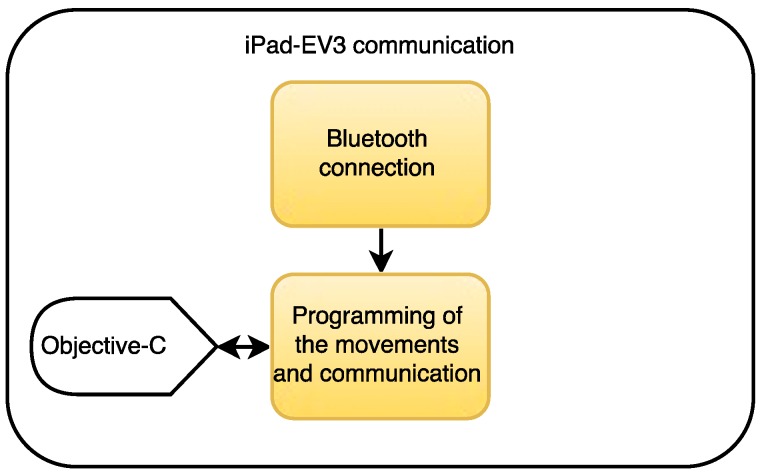
Communication protocol between the iPad and the EV3 brick.

The main purpose of this third stage is to design the communication between the iPad mini and the Lego EV3 brick. As shown in Figure 5, both devices are connected though a Bluetooth connection whose protocol is implemented in the iOS device through the XCode programming environment. 

The robot’s movement have also been implemented in the iOS program, by the use of the Objective-C programming language. These methods, allow the robot to move both its arms and legs when the activity is correctly completed and thus provide visual feedback and motivate the elderly people.

#### 2.1.6. Stage 4: Data Cloud Computing

The final step consists in uploading all the data to a server in order to make it accessible to the staff at the Santa y Real Casa de la Misericordia nursing home from a website.

**Figure 6:**
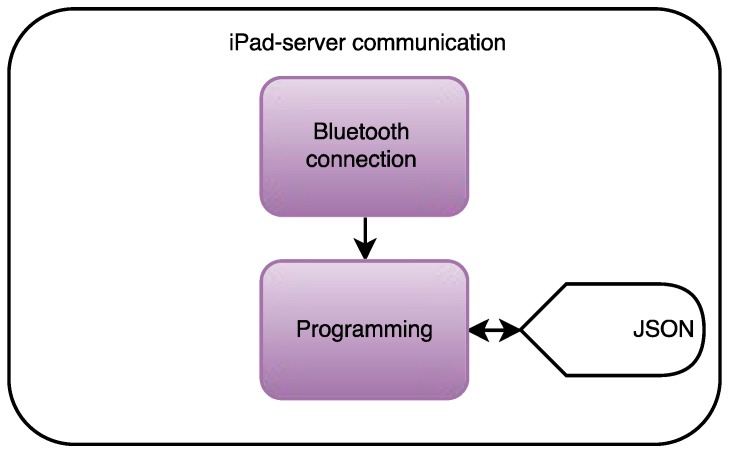
High-level design of the data cloud computing stage. JSON: JavaScript Object Notation

The high-level design of the communication between the iPad mini and the server in the fourth stage is shown in Figure 6. As shown in the figure, the data will be sent though JavaScript Object Notation (JSON) format “POST” requests. The server will process those JSON requests and will display the data on a website designed though the Django framework.

### 2.2. Materials

This section describes the hardware, software and sample used to conduct this experiment.

#### 2.2.1. Hardware

In the design, implementation and testing processes of this system, 6 different hardware components were used including an iPad mini tablet, the Lego Mindstorms Education kit, in addition to its EV3 brick, the MYO Electromyography (EMG) sensor and the Microsoft band sensor. The use of these hardware devices in the architecture shown in Figure 2 is remarkable and will be fully described in Section 4.

#### 2.2.2. Software

Firstly, the iOS application was programmed by means of the XCode software development kit (version 6.2, Apple Inc., Cupertino, CA, USA) and the Objective-C programming language. This iOS application, which is specially designed for iOS 9, provides the user with a brain reflexes serious game and is able to provide both cognitive and physical exercises. Furthermore, the application is capable of recording the scores and the heart rate value obtained for each user.

The iOS application is connected through the Bluetooth LE standard, using Apple’s “CoreBluetooth” framework and the “MicrosoftBand” framework for the two biological devices mentioned, the MYO and the Microsoft Band.

Moreover, in order to make the solution more user friendly and to give visual feedback, the iPad is connected to a Lego EV3 brick which is attached to a Lego structure with a humanoid appearance. Hence, the iPad will send specific commands to the Lego EV3 and this brick is capable of moving both arms and legs to give visual feedback when the user performs the activity correctly. These commands are programmed into the iOS application and are sent through the Bluetooth standard and interpreted by the EV3 brick.

Lastly, it must be mentioned that all the data related to each user are locally stored on the iPad using a SQLite relational database. Moreover, when the iPad has Internet access, all the data are uploaded to a server so that the data are stored and can be accessible to the nursing home staff through a website in order to monitor users’ data.

#### 2.2.3. Participants

The system was tested with a total of seven people (three men and four women) with an average age of 78.0 (Standard deviation (SD) = 7.75). All the patients testing the system were randomly selected from the Santa y Real Casa de la Misericordia nursing home in Bilbao. The subjects suffered different physical and cognitive impairments but none of them showed mental impairment.

This study was approved by the University of Deusto institutional ethics review board (Protocol registered No. 2015/0032).

### 2.3. Implementation

After having designed the system and selected the software and hardware to be used in the project, it was implemented. Implementation of the system took into account all the points stated in Section 2.1, which were based on the requirements of all the stakeholders.

**Figure 7:**
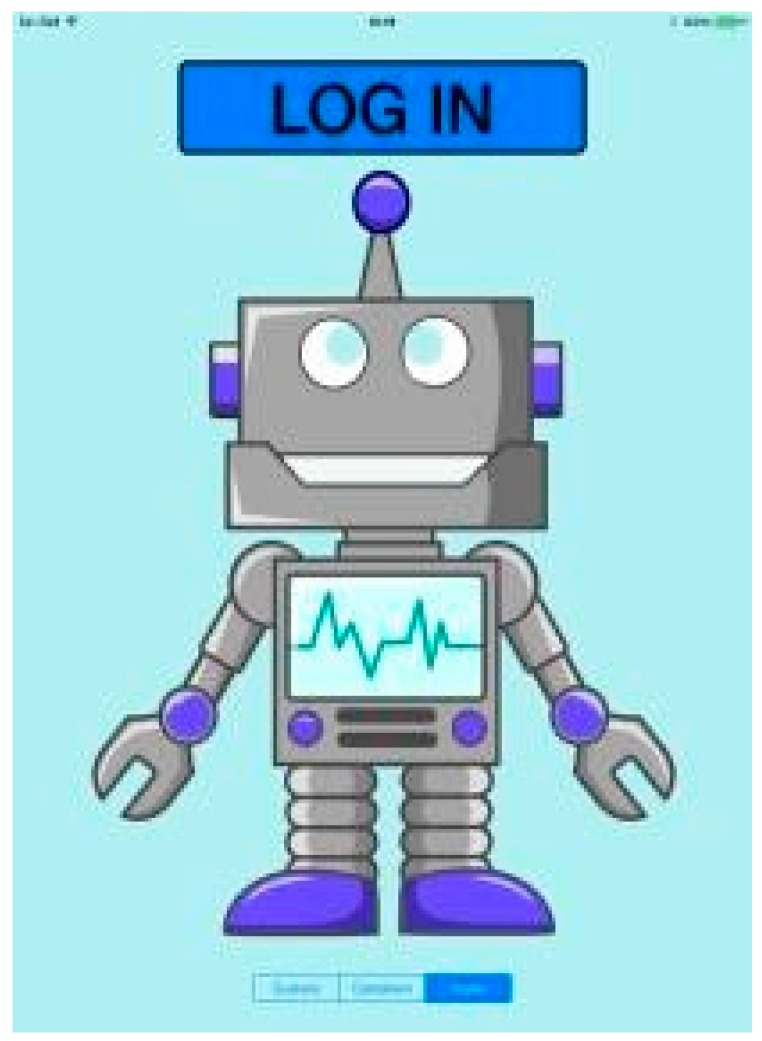
Log-in screen.

**Figure 8:**
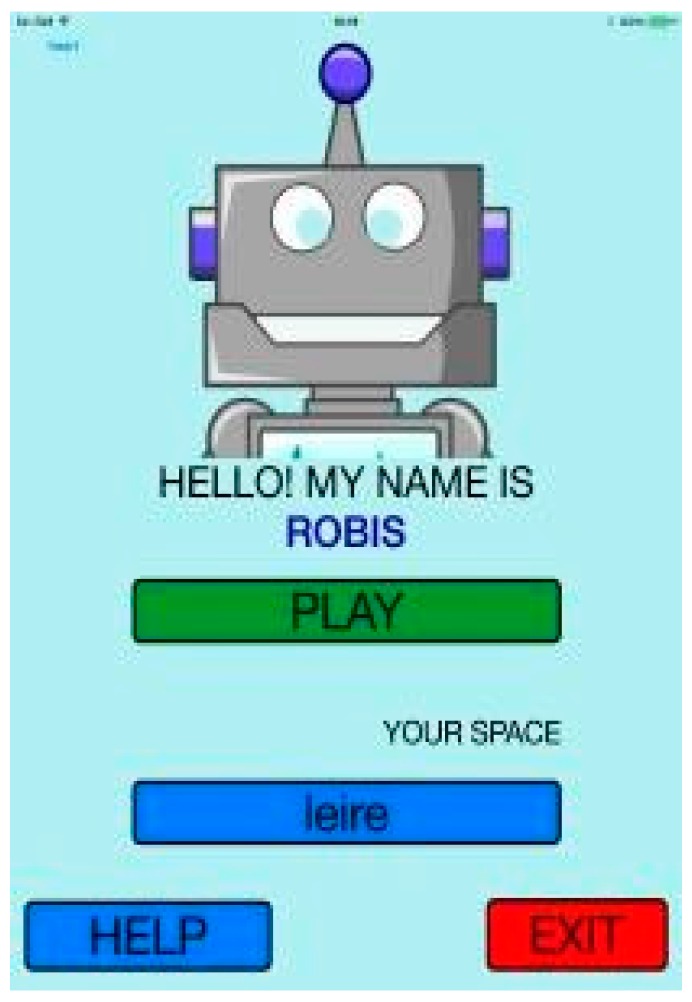
Graphical User Interface of the iOS application.

As mentioned in Section 2.1, the first point was to implement the GUI of the iOS application, which can be seen in Figure 7 and Figure 8.

While Figure 7 shows the log-in screen, Figure 8 shows the main user profile screen. In fact, as shown above, the buttons and text font used are big and clear so that elderly people have no difficulty reading and understanding them.

**Figure 9:**
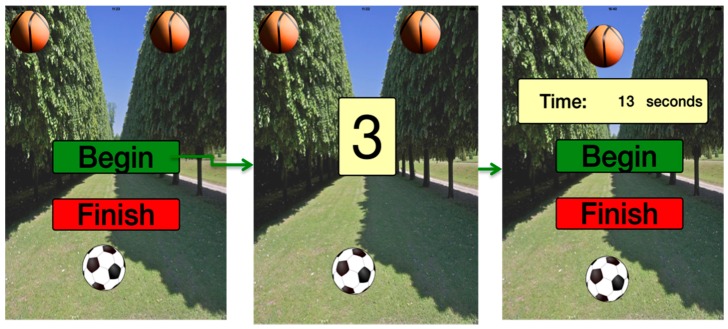
Designed serious game.

The biological parameters were gathered through the implementation of software based on the frameworks mentioned in Section 2.2 and the values were applied to the game shown in Figure 9.

**Figure 10:**
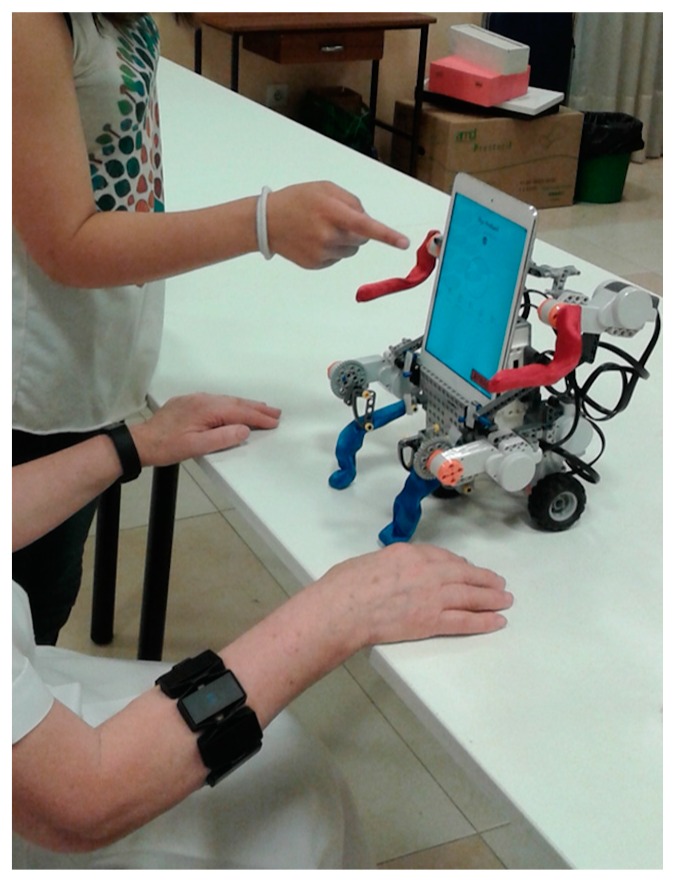
User interacting with the system.

After having integrated the Bluetooth communication between the EV3 brick and the iPad in order to perform the movements of the servomotors, the whole body of the robot was assembled, so that the servomotors could represent the user’s hands and legs. The final appearance of the whole system can be seen in Figure 10.

### 2.4. Experimental Protocol

All the participants followed the same assessment process, which consisted in performing a brain reflexes game, twice controlled by a MYO sensor and twice controlled by their fingers. After completing the task, the users were asked to fill in various questionnaires in order to perform a statistical assessment and evaluate the system. In order to do so, three different questionnaires were selected. First, an ICT use questionnaire, in order to determine to what extent the users were accustomed to using ICT devices; this questionnaire was built by the DeustoTech-Life Department and is based on the Questionnaire for User Interaction Satisfaction (QUIS) [23] standard questionnaire. Second, a Quality of life questionnaire (WHOQOL-BREF) [24], with the aim of making an international cross-culturally comparable quality of life assessment, in order to compare this system between different social situations, in the future. Last, a System Usability Scale (SUS) [25], which consists in 10 items with five response options, ranging from “strongly agree” to “strongly disagree” and is capable of measuring the usability of the developed software and hardware systems.

- In summary, the methodology applied to test the system can be described as follows:
- Location: the Santa y Real Casa de la Misericordia nursing home in Bilbao, Spain.
- Duration of the session: 25 min.
- Frequency: Once every 3 months.
- Exercise types: Cognitive and physical activities.
- Questionnaires:
  (1) WHOQOL-BREF
  (2) ICT Questionnaire
  (3) SUS

## 3. Results and Discussion

This section will analyze and present the feedback gathered from the trials and questionnaires that were conducted at the Santa y Real Casa de la Misericordia nursing home in Bilbao. Table 1 shows the results obtained from the SUS questionnaire. According to this questionnaire, the system is thought to be well-designed if the mean obtained is above 68. Hence, as shown in the Table 1, with a mean score of 79.29 points, the system is considered to be well-designed in terms of usability.

Table 2 shows the results obtained from the WHOQOL-BREF questionnaire regarding the quality of life of the end users living at the mentioned nursing home. As stated before in the Methodology section, this questionnaire will be used in future trials in order to carry out a cross-culturally comparable quality of life assessment. As this system has only been tested in Spain so far, these parameters have not been correlated with any of the parameters in this study. Hence, these scores have been taken as descriptive values of the sample. It must be noted that in this questionnaire the results of the mean value, minimum value and maximum value can range from 0 to 5, being 0 the worst option and 5 the best option. The meaning of each of the parameters of this questionnaire is as follows:

- WHOQOL DOM 1: physical health
- WHOQOL DOM 2: psychological domain
- WHOQOL DOM 3: social relationships
- WHOQOL DOM 4: environment

Table 3 shows the measurements obtained from the Microsoft band in terms of the heart rate, both at the beginning of the tests and at the end. From the results obtained, it can be observed that the heart rate value difference between the minimum value at the beginning and at the end does not change more than 10 bpm. This is the same for the case of the maximum heart rate value. This may show that the biofeedback system is properly implemented and the user is established.

The correlations between the total score obtained in the SUS questionnaire and the different major variables are shown in Table 4. From the table it can be seen that the scores obtained in the SUS questionnaire did not depend on how well the user performed the activity, both in the case of the MYO sensor and when not using the mentioned electromyography sensor. However, between the SUS and QUIS questionnaires, a strong correlation was detected in the information section (*R* = 0.683, *p* = 0.033). In fact, the higher scores users give the system in the SUS questionnaire, the better they rate the screen and information section on the QUIS questionnaires. This may mean that when the user is comfortable with the screen and feels that the information appearing on the application screen is clear and explicit, he or she will probably perceive that the application is, in fact, usable, which is one of the main objectives of a tablet application.

Some aspects that were detected during the testing phase are described in detail:

- The users from the Santa y Real Casa de la Misericordia nursing home were not familiar with ICTs. Hence, it has been noted that the scores obtained on the cognitive exercises improved after their initial contact with the system.
- Three of the seven users preferred playing without the MYO sensor. In fact, they preferred playing the game by controlling the ball with their finger. This was due to the fact that they were not accustomed either to using this kind of sensors or to controlling games with hand movements. In fact, elderly people consider putting external devices on their body to be too artificial. Therefore, the ideal system should provide the biofeedback in a totally unobtrusive mode.

It has been observed that the majority of the elderly people living in the nursing home were able to use this tool on their own, which could mean that this system has an acceptable usability level.

## 4. Conclusions

This section, will discuss the strengths and limitations that have been concluded from the results during the testing phase of the described system.

### 4.1. Strengths

On the one hand, the main strength of this project is the combination of biofeedback techniques with Lego robots and serious gaming with the aim of providing both cognitive and motor rehabilitation for elderly people.

On the other hand, this project provides elderly people with not only a cognitive and motor rehabilitation tool, but with a personalized tool for rehabilitation and monitoring. It is thus adapted to each user’s needs and cognitive and physical level.

### 4.2. Limitations and Perspectives

In order to improve to improve the implementation of the biofeedback system in terms of the heart rate parameters, an Electrocardiography (ECG) sensor could be included instead of the Microsoft band that is now being used. With the ECG sensor, the system could obtain the heart rate variability which, in fact, provides more accurate information than the heart rate value.

One of the main improvements that could be included in this system is automated data analysis through cloud computing techniques.

Furthermore, the inclusion of more biomedical sensors would be an excellent possibility for this project as the addition of sensors such as Galvanic Skin Response (GSR) sensors and stress sensors could improve the biofeedback module of the system.

## Figures and Tables

**Table 1:** Results of the System Usability Scale (SUS) questionnaire (*n* = 7).

	Mean *	SD	Minimum *	Maximum *
SUS	79.29	23.57	30.00	100.00

* Out of 100. SD: Standard deviation.

**Table 2:** Results obtained from the WHOQOL-BREF questionnaire (*n* = 7).

	Mean *	SD	Minimum *	Maximum *
WHOQOL DOM 1	3.25	0.73	2.14	4.29
WHOQOL DOM 2	3.71	0.46	2.83	4.33
WHOQOL DOM 3	3.54	0.79	2.33	4.50
WHOQOL DOM 4	4.10	0.44	3.75	4.88

* Out of 100.

**Table 3:** Measurements from the biological sensors (*n* = 7).

	Mean *	SD	Minimum *	Maximum *
HR at the beginning	78.00	19.31	63.00	120.00
HR at the end	77.14	14.68	68.00	110.00

* Out of 100. HR: Heart rate.

**Table 4:** Correlation between SUS questionnaire and other variables.

	Correlation Coefficient (*R*)	*p*-Value
Game with MYO	0.098	0.761
Game without MYO	−0.429	0.176
QUIS (general impression)	0.617	0.06
QUIS (screen section)	0.293	0.362
QUIS (information section)	0.683 *	0.033

* Significant value (*p* < 0.05). QUIS: Questionnaire of User Interaction Satisfaction.
